# Multi-omics analysis reveals substantial linkages between the oral-gut microbiomes and inflamm-aging molecules in elderly pigs

**DOI:** 10.3389/fmicb.2023.1250891

**Published:** 2023-09-15

**Authors:** Chuanmin Qiao, Maozhang He, Shumei Wang, Xinjie Jiang, Feng Wang, Xinjian Li, Shuyi Tan, Zhe Chao, Wenshui Xin, Shuai Gao, Jingli Yuan, Qiang Li, Zichun Xu, Xinli Zheng, Jianguo Zhao, Guangliang Liu

**Affiliations:** ^1^Institute of Zoology, Chinese Academy of Sciences, Beijing, China; ^2^Institute of Animal Science and Veterinary Medicine, Academy of Agricultural Sciences, Haikou, China; ^3^Hainan Yazhou Bay Seed Laboratory, Sanya, China; ^4^Department of Microbiology, School of Basic Medical Sciences, Anhui Medical University, Hefei, China

**Keywords:** swine, aging, oral-gut axis, multi-omics, inflammation

## Abstract

**Introduction:**

The accelerated aging of the global population has emerged as a critical public health concern, with increasing recognition of the influential role played by the microbiome in shaping host well-being. Nonetheless, there remains a dearth of understanding regarding the functional alterations occurring within the microbiota and their intricate interactions with metabolic pathways across various stages of aging.

**Methods:**

This study employed a comprehensive metagenomic analysis encompassing saliva and stool samples obtained from 45 pigs representing three distinct age groups, alongside serum metabolomics and lipidomics profiling.

**Results:**

Our findings unveiled discernible modifications in the gut and oral microbiomes, serum metabolome, and lipidome at each age stage. Specifically, we identified 87 microbial species in stool samples and 68 in saliva samples that demonstrated significant age-related changes. Notably, 13 species in stool, including *Clostridiales bacterium*, *Lactobacillus johnsonii*, and *Oscillibacter* spp., exhibited age-dependent alterations, while 15 salivary species, such as *Corynebacterium xerosis*, *Staphylococcus sciuri*, and *Prevotella intermedia*, displayed an increase with senescence, accompanied by a notable enrichment of pathogenic organisms. Concomitant with these gut-oral microbiota changes were functional modifications observed in pathways such as cell growth and death (necroptosis), bacterial infection disease, and aging (longevity regulating pathway) throughout the aging process. Moreover, our metabolomics and lipidomics analyses unveiled the accumulation of inflammatory metabolites or the depletion of beneficial metabolites and lipids as aging progressed. Furthermore, we unraveled a complex interplay linking the oral-gut microbiota with serum metabolites and lipids.

**Discussion:**

Collectively, our findings illuminate novel insights into the potential contributions of the oral-gut microbiome and systemic circulating metabolites and lipids to host lifespan and healthy aging.

## 1. Introduction

The escalating number of elderly individuals is an escalating public health concern globally, with a particular emphasis on China, where it is estimated that there will be around 402 million older adults by 2040 (Wang and Chen, 2022). The swift aging of the population, stimulated by recent declines in fertility and mortality rates, spark concerns regarding the health and quality of life of elderly individuals, and will pose significant challenges for the healthcare system. The process of aging is intricate and dynamic, involving the restructuring of various physiological systems that are intricately associated with systemic inflammation, metabolism, and immunity across diverse cells and tissues (Fulop et al., 2017; Finger et al., 2022). However, the process of aging and lifespan is tremendously influenced by host genetics and environmental factors. The symbiotic commensal microbiota has been recognized as an influential environmental factor in the development of aging-related metabolic and immune diseases. Accumulating evidence supports the role of microbiota in the development of these diseases (Belkaid and Hand, 2014; Thaiss et al., 2016; Zheng et al., 2020; Ansaldo et al., 2021), and further research is needed to better understand the relationship between the microbiome and aging.

Altered gut microbiome and host metabolism have been implicated in the process of aging (Cruz-Pereira et al., 2022; Ghosh et al., 2022). Aging is associated with changes in the gut microbiota, which in turn can affect host metabolism (Gao et al., 2018). The gut microbiota is a complex community of microorganisms that live in the gastrointestinal tract and play an important role in maintaining human health (Thursby and Juge, 2017; Gomaa, 2020). As we age, the diversity and composition of the gut microbiota can change, with a decrease in beneficial bacteria and an increase in harmful bacteria. These changes in the gut microbiota can contribute to a number of age-related health problems, such as impaired immune function, inflammation, and metabolic dysfunction (Kong et al., 2019; Badal et al., 2020; Ghosh et al., 2022; Yin et al., 2023). For example, alterations in the gut microbiota have been linked to age-related diseases such as type 2 diabetes, cardiovascular disease, and cognitive decline (Pascale et al., 2019; Ghosh et al., 2020; Pellanda et al., 2021). The gut microbiome plays a critical role in host metabolism through a variety of mechanisms, including fermentation of dietary fibers, regulation of intestinal barrier function, regulation of immune function and bile acid metabolism, for instance, microbial derived SCFAs can modulate various metabolic pathways in the host, including glucose and lipid metabolism, and can also affect immune function and inflammation (Gasaly et al., 2021; Ohtani and Hara, 2021; Zhang, 2022). Overall, the relationship between the gut microbiota and host metabolism is complex and their joint action on aging still not fully understood. However, there is growing evidence to suggest that interventions aimed at modulating the gut microbiota, such as dietary changes or probiotics, may have potential therapeutic benefits for age-related metabolic disorders.

The study of aging and the host microbiome is a relatively new field of research. Though there have been many studies of the human microbiome and aging, there are still several deficiencies that need to be addressed since human gut microbiota extremely dynamic and influenced by a number of confounding factors, such as diet, medications, lifestyle factors, which can make it difficult to isolate the effects of aging on the gut microbiota (Bana and Cabreiro, 2019; Manor et al., 2020; Molinero et al., 2023). Further, many studies of the human, and others animal microbiome and aging focus on taxonomic changes, but do not investigate functional changes in the microbiome. Functional studies are needed to better understand the mechanisms by which the human microbiome influences aging-related processes. Besides, scarce researches investigate the process of aging using multi-omics, which involved metagenomics, metabolomics, lipidomics, and transcriptomics to provide a more comprehensive understanding of biological systems that are involved in the aging process. By integrating these different types of data, researchers can identify key molecular and cellular changes that occur during aging, and can use this information to develop new approaches for preventing or treating age-related diseases. In addition, previous researches mainly focus on the role of gut microbiota in the host aging. However, the importance of the oral microbiome in the aging process is increasingly recognized, as the oral microbiome plays a key role in maintaining oral health and is also implicated in various systemic diseases (Sedghi et al., 2021; Peng et al., 2022).

Hence, a well-controlled model system that reproduces faithfully the trajectories in the oral and gut microbiota with age is warranted and will provide a better understanding of the role played by them in the healthy development and aging of the host. Pigs are used as an excellent model to study the interaction between host microbiome and aging by combining multi-omics, because pigs share many similarities with humans in terms of their anatomy, physiology, and nutritional requirements. For example, the structure and function of the pig gut is similar to that of humans, as well as in organ development and disease progression (Lunney et al., 2021; Rose et al., 2022). In addition, swine can be raised in a controlled environment and are readily available and relatively inexpensive compared to other animal models, which allows researchers to manipulate their diet and other environmental factors that may influence the host microbiome and aging process. Previous research has also been identified that pigs have a gut microbiome that is similar in composition to that of humans, with a high degree of microbial diversity and similar microbial taxa (Lim et al., 2019; Yang et al., 2022). Overall, the use of pigs as a model for studying the interaction between host microbiome and aging provides a valuable tool for understanding the complex interplay between these factors, and for developing interventions and therapies that can improve healthspan and reduce the burden of age-related diseases in humans.

**Figure 1:**
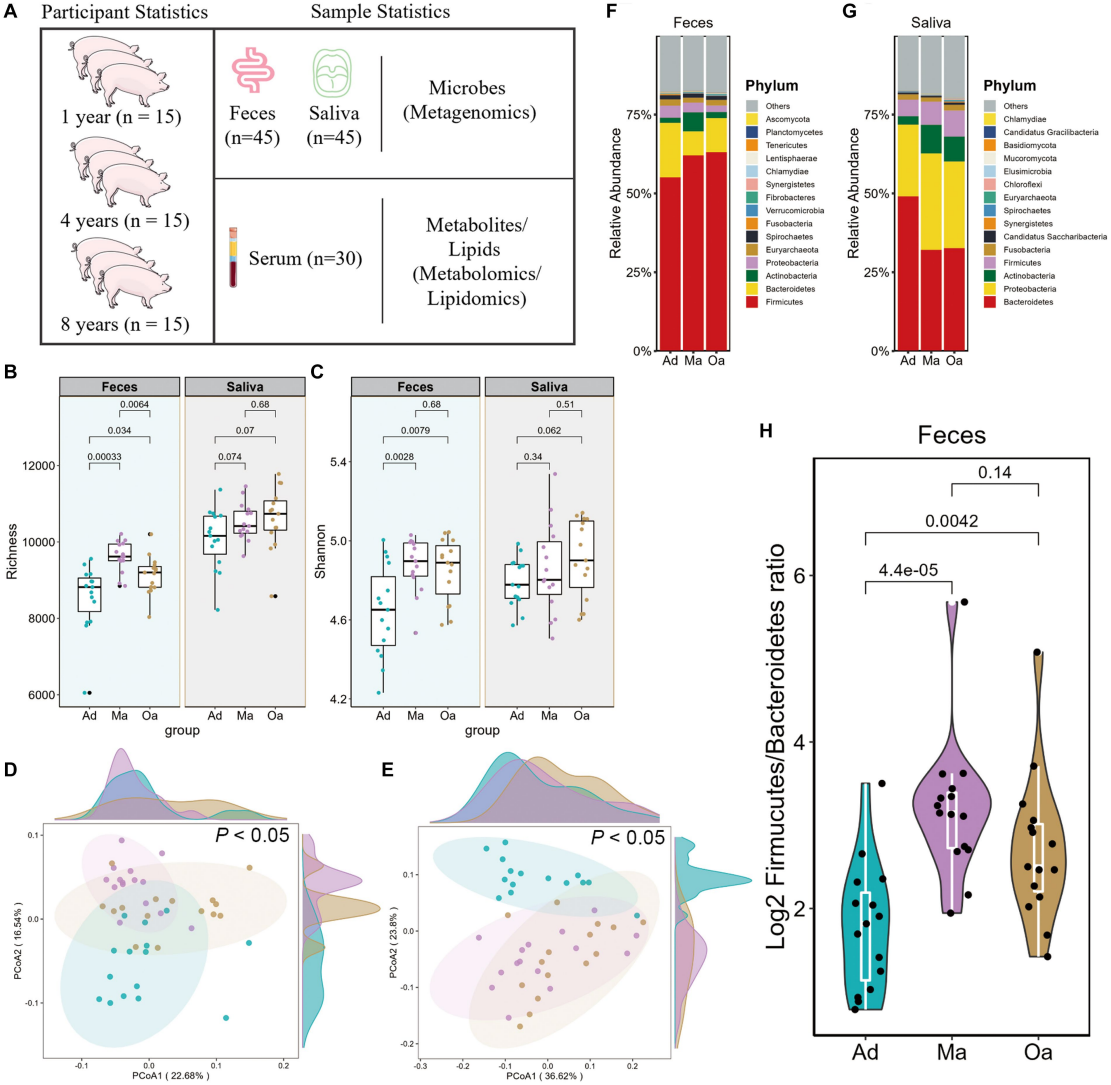
Alterations of gut microbial composition in relation to aging in pigs. **(A)** Workflow overview of the metagenomic, metabolomic, and lipidomics strategies used in this study. Comparison of α-diversity by **(B)** richness and **(C)** Shannon indices using Wilcoxon rank sum test in pigs at adult (Ad), middle age (Ma), and old age (Oa). **(D,E)** Principal coordinate analysis (PCoA) based on the Bray–Curtis dissimilarity metric derived from feces and saliva showed a significant difference in gut and salivary microbial composition among Ad, Ma, and Oa groups. Statistical significance and variance of Bray–Curtis dissimilarity data were assessed using PERMANOVA. **(F,G)** Relative abundance of bacterial phyla in gut and oral microbiotas of individuals with Ad, Ma, and Oa. **(H)** Log_2_ Firmicutes/Bacteroidota ratios in gut samples from individuals in Ad, Ma, and Oa groups (Wilcoxon rank-sum test).

To date, there is a paucity of data investigating the progression of aging through the integration of metagenomics analyses of oral and gut microbial species, with metabolomics and lipidomics profiling of blood molecules. In this study, we recruited a cohort of 45 pigs and collected 45 fecal and 45 salivary samples, as well as 30 blood samples at three different age points: (1) 1 year old, (2) 4 years old, and (3) 8 years old (Tohyama and Kobayashi, 2019). The use of this animal model allowed us to explore the potential correlations among these factors and their possible roles in the observed changes associated with aging (Figure 1A). Our primary objective was to determine the complex molecular changes that occur at different stages of age in pigs, and how they contribute to age-related alteration in host metabolism and decline in physiological functions. Multi-omics approaches can provide a more comprehensive understanding of the aging process, as different types of molecules are interconnected and influence each other in complex ways. By analyzing multiple omics data sets, we can identify molecular signatures and pathways that are associated with aging and age-related diseases. This can lead to the development of new biomarkers and therapeutic targets for age-related conditions, as well as strategies for promoting healthy aging and extending lifespan. In general, the study of aging by multi-omics is an important and rapidly growing field that has the potential to revolutionize our understanding of aging and age-related diseases, and ultimately improve human health and well-being.

## 2. Materials and methods

### 2.1. Study design and sample collection

A cohort of 45 pigs was enrolled in this study, and fecal and salivary samples were collected from each pig. The pigs were categorized into three age groups, with 15 pigs in each group: one-year-old pigs, those approximately 4 years old, and those approximately 8 years old. Additionally, serum samples were randomly collected from 10 pigs in each age group. This study design allowed for the collection of comprehensive and representative biological samples across different age points, which is essential for investigating the complexities of the aging process.

### 2.2. Shotgun metagenomic sequencing and analysis

In this study, the microbial alterations induced by age were determined by extracting DNA from saliva and fecal samples using QIAamp DNA mini kit (QIAGEN, Germany). Shotgun metagenomic sequencing of porcine saliva and fecal DNA was performed on an Illumina HiSeq 2000 platform (Illumina, United States). To process the raw sequence reads, Trimmomatic (version 0.39) (Bolger et al., 2014) was used to remove low-quality reads and adaptors. The resulting trimmed reads were aligned to the porcine genome (*sus scrofa*, Sscrofa11.1) using Bowtie 2 (version 2.4.4) (Langmead and Salzberg, 2012) to remove host reads. The non-swine reads were assembled using MEGAHIT (v.1.1.3) with default parameters (Li et al., 2015), and taxonomy classification was performed using Kraken2 (version 2.0.8). Bracken was utilized to improve species-level abundance estimation based on Kraken2 results (Wood et al., 2019). Functional annotation of genes was performed by aligning to KEGG database using DIAMOND (v.0.9.32.133) (Buchfink et al., 2015), and the best-hit with identity ≥30% and coverage ≥70% was selected. The Chao1 and Shannon indices were calculated using the R packages picante and vegan based on the microbial abundance matrix at the species level. The dissimilarity matrix constructed using the Bray–Curtis method was employed to visualize the differences in microbiota composition between different groups.

### 2.3. Chemicals and regents

Sigma Aldrich provided Ammonium acetate (NH_4_AC), Merck provided Acetonitrile, and Fisher provided ammonium hydroxide (NH_4_OH) and methanol. Thermo Fisher provided MS-grade methanol, MS-grade acetonitrile, and HPLC-grade 2-propanol. Sigma provided HPLC-grade formic acid and HPLC-grade ammonium formate. All chemicals used in this study were of high purity and quality, ensuring the reliability and reproducibility of the results.

### 2.4. Analysis of plasma metabolomics

Untargeted metabolomic and lipidomic profiles of fasting serum and stool samples were measured by combining two UHPLC-MS/MS methods, including metabolites in both positive and negative ionization modes. The raw data files were processed using Compound Discoverer 3.1 (CD3.1, Thermo Fisher) to perform peak alignment, peak picking, and metabolite quantitation. The main parameters included: a retention time tolerance of 0.2 min; actual mass tolerance, 5 ppm; signal intensity tolerance of 30%; signal/noise ratio of 3; and minimum intensity of 100,000. Peak intensities were normalized to the total spectral intensity and used to predict the molecular formula based on additive ions, molecular ion peaks and fragment ions. Peaks were then matched with the mzCloud,^1^ HMDB, mzVault and MassList databases for untargeted metabolomic analysis, and with the Lipidmaps and Lipidblast databases for lipidomic analysis. Accurate qualitative and relative quantitative results were thus obtained. Partial least squares discriminant analysis (PLS-DA) was used to reveal the metabolites changes in groups by R package *ropls* (Thevenot et al., 2015) and the abundance of significant metabolites with variable important in projection (VIP) >1 and with corrected *p*-value (Wilcoxon test) <0.05 were selected for enrichment analysis. The enrichment pathway of differential plasma metabolite profile between any two groups in pigs was analyzed by MetaboAnalyst 5.0^2^ (Pang et al., 2022), respectively.

### 2.5. Sample preparation for lipidomics analyses

For the purpose of non-targeted lipid profiling, lipids were isolated from plasma samples using established techniques, as described previously (Zhou et al., 2017). Briefly, a 200 μL volume of water was added to sample and vortexed for 5 s. Subsequently, 240 μL of precooling methanol was added and the mixture vortexed for 30 s. After that, 800 μL of MTBE was added and the mixture was ultrasound 20 min at 4°C followed by sitting still for 30 min at room temperature. The solution was centrifuged at 14,000 g for 15 min at 10°C and the upper organic solvent layer was obtained and dried under nitrogen. Reverse phase chromatography was selected for LC separation using CSH C18 column (1.7 μm, 2.1 mm × 100 mm, Waters). The lipid extracts were re-dissolved in 200 μL 90% isopropanol/acetonitrile, centrifuged at 14,000 g for 15 min, finally 3 μL of sample was injected. Solvent A was acetonitrile–water (6:4, v/v) with 0.1% formic acid and 0.1 mM ammonium formate and solvent B was acetonitrile–isopropanol (1,9, v/v) with 0.1% formic acid and 0.1 mM ammonium formate. The initial mobile phase was 30% solvent B at a flow rate of 300 μL/min. It was held for 2 min, and then linearly increased to 100% solvent B in 23 min, followed by equilibrating at 5% solvent B for 10 min. Mass spectra was acquired by Q-Exactive Plus in positive and negative mode, respectively. ESI parameters were optimized and preset for all measurements as follows: source temperature, 300°C; capillary temperature, 350°C, the ion spray voltage was set at 3000 V, S-Lens RF Level was set at 50% and the scan range of the instruments was set at m/z 200–1800. “Lipid Search” is a search engine for the identification of lipid species based on MS/MS math. Lipid Search contains more than 30 lipid classes and more than 1,500,000 fragment ions in the database. Both mass tolerance for precursor and fragment were set to 5 ppm.

### 2.6. Statistical analysis

All statistical analyses were conducted in R platform (version 4.0). For statistic in multiple groups, we utilized Kruskal–Wallis one-way ANOVA to evaluate the difference among three groups. Only the remarkably different indices in three groups were evaluated by further Mann–Whitney *U* test with Bonferroni correction as post-hoc test between each of two groups. Values of adjusted *p*-value less than 0.05 were considered statistically significant. Error bars indicate mean ± standard error (se). The Mfuzz package in R to conduct the cluster analysis. Spearman’s rank correlation coefficients were calculated and corrected for multiple testing using the Benjamini–Hochberg method. For the correlations between differentially microbial species, KO genes, metabolites, and lipidomics, a significance threshold of 0.05 and an absolute correlation threshold of 0.6 were applied.

## 3. Results

### 3.1. Description of the study

In the present research, we performed a multi-omic oral and gut microbiome study of pigs at 1 year old, 4 years old, and 8 years old. This extended previous studies that based on 16S rRNA gene sequencing and limited in gut region. This study additionally includes metagenomics analyses of the oral cavity, metabolomics, and lipidomics of serum in tested subjects (Figure 1A). We generated metagenomic data for 45 faeces and 45 saliva, while metabolomics, and lipidomics data for 30 serum samples, respectively. In sum, a total of 575.6 Gbp of DNA sequencing data was acquired.

Across all samples with available metagenomic data, the total clean DNA sequencing data amounted to 292.0096 Gbp for fecal specimens and 282.64 Gbp for sputum samples. Each sample yielded an average of 6.49 Gbp for feces and 6.28 Gbp for saliva. For serum samples, we identified 1,150 metabolites in positive ion mode and 496 in negative ion mode, and 2,402 lipids through lipidomics. By integrating these multi-omics datasets, we provide a comprehensive analysis of the changing trajectory of aging-related gut-oral microbial species, functional genes, and pathways, along with their intimate correlations with serum metabolites and lipids.

### 3.2. Increased alpha-diversity and altered overall saliva-faeces microbial composition in swine across different stages of age

We performed metagenomic analyses in pigs with nearly 1 year old (adult, Ad group), 4 years old (middle age, Ma) and 8 years old (old age, Oa) to explore the link between the oral-gut microbiome and different stage of ages. In faeces, Chao1 index showed significant difference among the porcine age groups, while there was no significant alteration in Shannon’s diversity index between Ma and Oa groups except between Ad and Ma groups. However, in saliva samples, both alpha diversity indexes showed no statistical difference among these three groups, though consistently exhibited higher alpha diversity from Ad to Oa stage (Figures 1B,C). Furthermore, principal coordinate analysis (PCoA) based on Bray–Curtis distances from gut and oral microbiota at species showed significant separation among the three groups (*p* < 0.05; Figures 1D,E). These results all suggested that age played a great role in the composition of oral and gut microbiota of swine.

At the phylum level, we observed considerable differences in the oral and gut microbial profiles among the Ad, Ma and Oa groups. In the stool sample, the relative abundance of Firmicutes was increased in parallel with age, whereas Bacteroidetes was more abundant in the Ad group and Actinobacteria was more abundant in the Ma group (Figure 1F). In the saliva sample, we observed the relative abundance of Bacteroidetes decreased with aging. Inversely, Proteobacteria and Actinobacteria increased in the Ma and Oa groups as compared with Ad group (Figure 1G). In addition, the Firmicutes/Bacteroidetes (F/B) ratio was a measure of the relative abundance of Firmicutes and Bacteroidetes bacteria in the gut microbiome. It has been suggested that this ratio may be a useful indicator of overall gut health and may also be associated with various health conditions, we compared the ratio in the three groups. The relative abundance of Firmicute was higher in subjects of Ma and Oa groups than in the Ad group, whereas the proportion of Bacteroidetes was lower in Ma group, accordingly, we found a higher Firmicutes/Bacteroidetes ratio in the pigs of Ma group than in the Oa and Ad groups (Figure 1H).

### 3.3. Age-dependent taxonomic signatures of oral and gut microbial species

**Figure 2:**
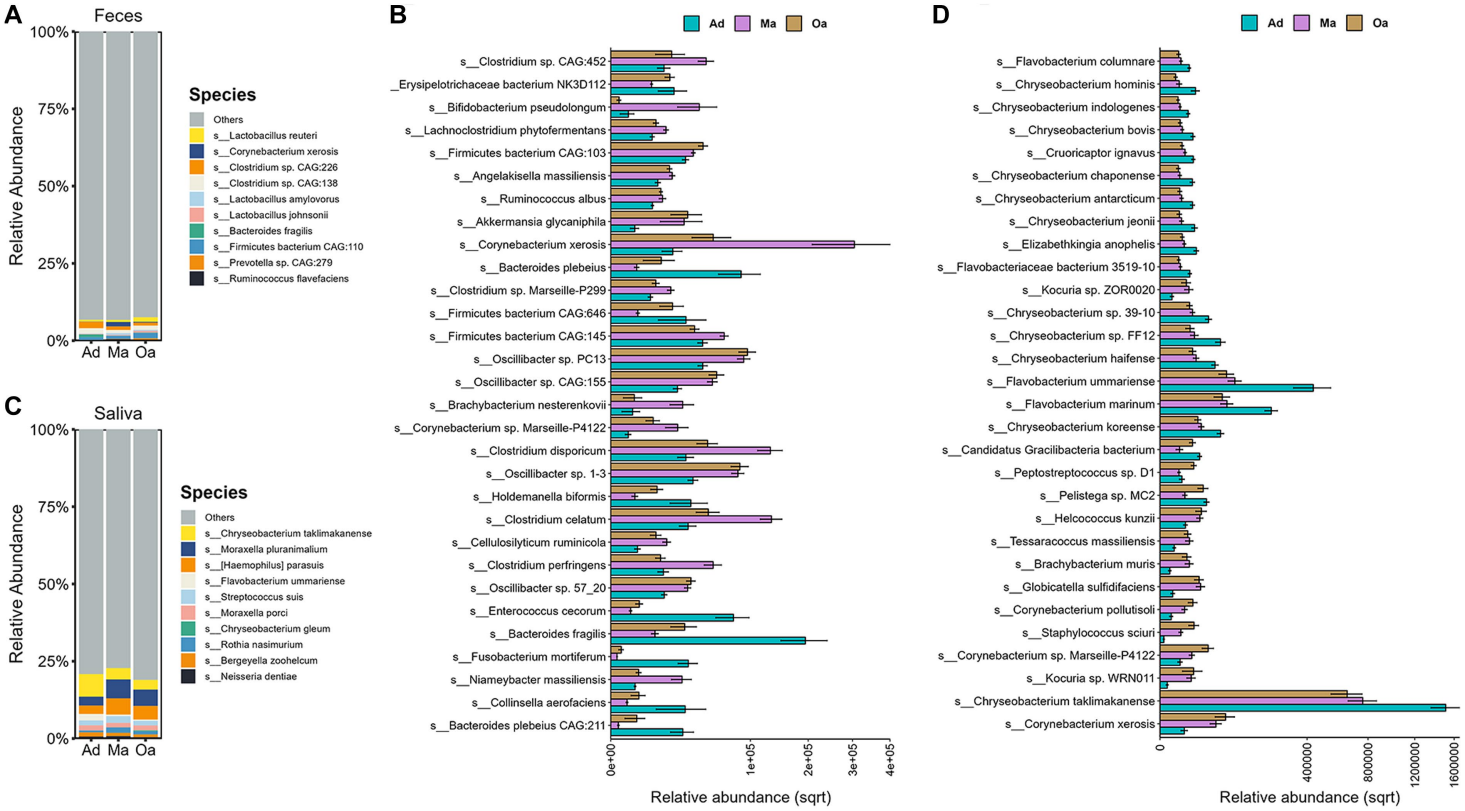
Taxonomic signatures of oral and gut microbial species showed age-dependent. **(A)** Stacked bar chart displays the top 10 most abundant microbes at species level in stool sample among the three groups. **(B)** The Kruskal–Wallis test was employed to identify microbes that differed significantly among three groups (with corrected *p*-value <0.05). The grouping error bar plot displays the top 30 microbes with the largest differences in stool samples. **(C)** The stacked bar chart displays the top 10 most abundant microbes in different groups of saliva samples, ranked by their relative abundances. **(D)** The Kruskal–Wallis test was employed to identify microbes that differed significantly among three groups (with corrected *p*-value <0.05). The grouping error bar plot displays the top 30 microbes with the largest differences in salivary samples.

Previous investigations have primarily relied on 16S rDNA microbiome profiling, which has imposed limitations on the comprehensive assessment of age-related variations in the abundance of microbial species and their functional capacities within the microbial community. In the present study, our objective was to investigate the alterations in compositions and functions of the oral-gut axis microbiota associated with age. To achieve this, we employed shotgun metagenomics to analyze sputum and fecal specimens obtained from swine, enabling a more in-depth exploration of the microbial community. Furthermore, we conducted a comparative analysis of microbial abundance across different age groups to elucidate the potential impact of age on the oral-gut axis microbiota. Among the top 10 species in the Ad, Ma, and Oa groups, the most abundant in the gut were *Lactobacillus reuteri*, *Lactobacillus johnsonii*, *Lactobacillus amylovorus* (each belonging to the genus *Lactobacillus*), *Ruminococcus flavefaciens*, *Bacteroides fragilis*, and *Corynebacterium xerosis* (Figure 2A). Next, we determined differentially abundant species among the three groups and between any two groups by using Kruskal–Wallis and Wilcoxon rank-sum test analyses, respectively. Furthermore, we only considered those species that make up at least 0.05% of the relative abundance of the entire community and have adjusted *p*-values <0.05. Examination of the microbiota at the species level identified a consortium of bacteria that were significantly altered by the effect of age. When comparing the top 30 different species in each group, the majority of only Ad-enriched bacteria were from genus Bacteroides, including *Bacteroides fragilis, Bacteroides plebeius CAG:211*, and *Bacteroides plebeius*. On the other hand, the abundances of several species from genus Clostridium, comprising *Clostridium perfringens, Clostridium celatum, Clostridium disporicum*, as well as *Bifidobacterium pseudolongum* were notably enhanced in Ma group. What’s more, the abundances of *Oscillibacter* sp. *57_20 s*, *Oscillibacter* sp. *CAG:155*, and *Oscillibacter* sp. *1–3* were also found increased with age (Figure 2B and Supplementary Table S1). Further, pairwise comparisons identified 62 microbial species that showed differential expression between Ma and Ad groups, 36 species between Oa and Ad groups, and 43 species between Oa and Ma groups (Supplementary Figures S1A–C and Supplementary Table S2).

We next investigated which salivary microbes show significant differences across age. We observed *Chryseobacterium taklimakanense*, *Moraxella pluranimalium, Flavobacterium ummariense,* and *Moraxella porci* were the top abundant species in the saliva samples of pigs in Ad, Ma, and Oa groups (Figures 2C). In particular, our results show a number of taxa that increase with age, including the species *Corynebacterium xerosis, Staphylococcus sciuri, Actinobacillus seminis, Escherichia coli*, and *Corynebacterium pollutisoli*, or decrease with age, including the species *Flavobacterium marinum, Flavobacterium ummariense*, *Chryseobacterium bovis*, and *Chryseobacterium hominis* (Figure 2D; Supplementary Figures S1D–F and Supplementary Table S3). In addition, a total of 66 salivary microbes were found as significantly different between Ma and Ad groups, 66 species between Oa and Ad groups, and 8 microbes between Oa and Ma groups (Supplementary Figures S1D–F and Supplementary Table S4).

### 3.4. Age-specific serum metabolomic and lipidomic features in domestic pigs

**Figure 3:**
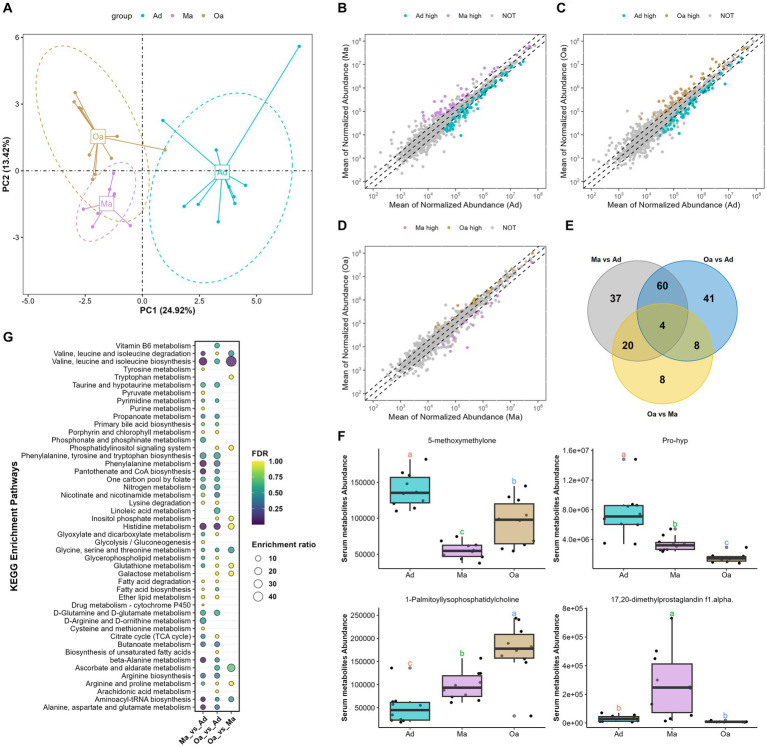
Aging altered the overall serum metabolites composition. **(A)** Principal component analysis (PCA) of serum metabolomics data revealed a significant deviation in metabolite composition among different age groups. **(B)** Compared Ma with Ad group, scatter plot showed that 44 metabolites with significantly higher abundance in Ma group are indicated with purple, while the 77 metabolites with significantly higher abundance in Ad group are in cyan. **(C)** Scatter plot showed that 48 metabolites with significantly higher abundance in Oa group are indicated with brown, while the 65 metabolites with significantly higher abundance in Ad group are in cyan. **(D)** Scatter plot showed that 22 metabolites with significantly higher abundance in Oa group are indicated with brown, while the 18 metabolites with significantly higher abundance in Ma group are in purple. **(E)** The Venn diagram illustrated the intersection based on significant differential metabolites derived from any two groups, and revealing that 4 metabolites were present in the differential results of all 3 pairwise comparisons. **(F)** The box plot displays the relative abundances of intersecting 4 metabolites across different age groups, with letters above the plot indicating significant differences between groups. Different letters between two groups indicate significant differences. **(G)** A metabolic pathway analysis was performed based on metabolites showing differences between pairwise groups. The size of the circles represents the degree of pathway enrichment, while the color of the circles indicates the significance of the pathway.

Serum metabolites and lipids are known to play a key role in mediating the metabolic and immune interactions between the microbiome and its host, thus providing a fundamental view into the complex dynamics of host age and physiology. To refine the metabolomic and lipidomic features across age, we performed untargeted metabolomic and lipidomic profiling by UHPLC-MS/MS analysis. A subset of 30 participants (each 10 pigs from Ad, Ma, and Oa groups) from this study was included in the serum metabonomic and lipidomic study. In serum samples, 1,646 known metabolites and 2,402 lipids were yielded. Both qualitative PCA and PLS-DA analyses were performed to evaluate metabolomic and lipidomic composition. From the prospective of serum metabolites, overall metabolic abundance of swine at different stages of age was different from that of each other as indicated by PCA analysis (Figures 3A *p* < 0.05, PERMANOVA test) and PLS-DA analysis (Supplementary Figure S2A). To identify significantly altered metabolites that may be important across the stages of age, we performed pairwise comparisons between groups. When Ma was compared with Ad, a sum of 121 metabolites were significantly altered (Figure 3B). These include the enrichment of deoxycholic acid, 1-palmitoylphosphatidylcholine, 1-methylhistidine, lipoxin a4, phenaceturic acid, and 1h-indole-3-propanoic acid. In contrast, succinate, taurine, leucine, L-isoleucine, and glutamic acid was depleted in Ma pigs compared with Ad (Supplementary Figure S2B and Supplementary Table S5). With the comparison of Oa with Ad, 113 metabolites were detected differentially abundant (Figure 3C), including the depletion of taurine, succinate, phenylalanine, glutamic acid and folinic acid in Oa. Particularly, deoxycholic acid, salicylic acid, phenaceturic acid, chenodeoxycholate, 1-palmitoyllysophosphatidylcholine, hippuric acid, and valine betaine were enriched in Oa and Ma when both compared to Ad subjects (Supplementary Figure S2C and Supplementary Table S6). Moreover, 40 metabolites were identified with differential expression in Oa and Ma groups. Notably, sarcosine, 1-palmitoyl-lysophosphatidylcholine and histamine were found to show increasing trends from Ad, through Ma, to Oa (Figure 3D; Supplementary Figure S2D and Supplementary Table S7). Among them, four metabolites (5-methoxymethylone, pro-hyp, 1-palmitoyl-lysophosphatidylcholine, and 17,20-dimethyl prostaglandin F1alpha) overlapped with those of aging correlations (Figures 3E,F), suggesting their potential contribution to the progression of different stages of age. Furthermore, MetaboAnalyst was used for searching KEGG database to explore the most relevant metabolic pathways based on differential metabolites from each pair of inter-group differential analyses. Valine, leucine and isoleucine biosynthesis, and Histidine metabolism were significantly influenced by age (Figure 3G).

**Figure 4:**
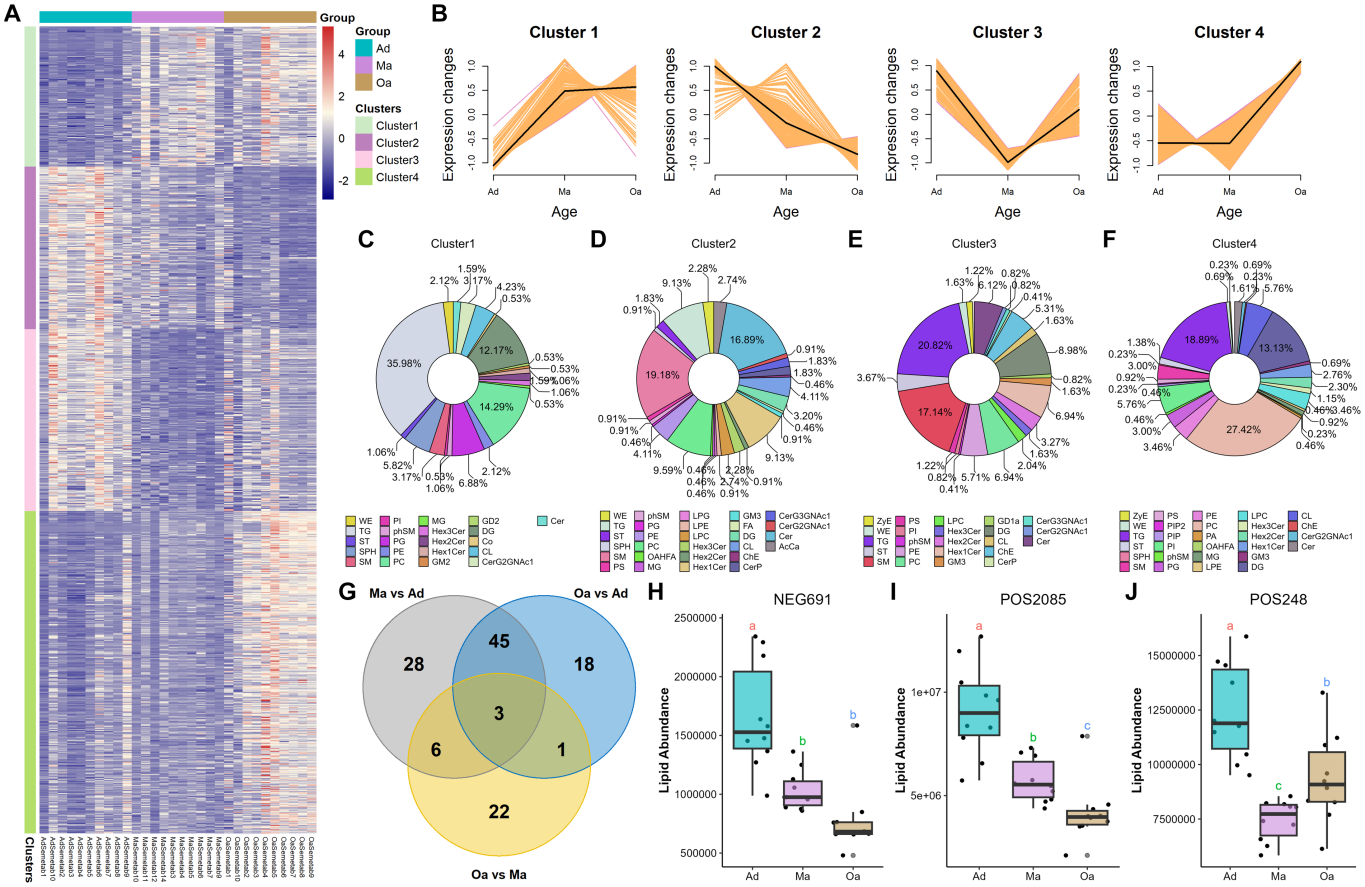
Significant differences in lipid profiles among different age groups. **(A)** The differentially altered lipids (DALs) that showed significant changes were clustered and represented in a heatmap. **(B)** Four clusters (Clusters 1–4) were highlighted, and the trend lines indicating the change in serum levels of these differentially altered lipids (DALs) were displayed. **(C–F)** The differentially altered lipids (DALs) in each cluster were further subcategorized based on their lipid classes. **(G)** The Venn diagram illustrated the intersection based on significant differential lipids derived from any two groups, and revealing that 3 lipids were present in the differential results of all 3 pairwise comparisons. **(H–J)** The box plot displays the relative abundances of intersecting 3 lipids across different age groups, with letters above the plot indicating significant differences between groups. Different letters between two groups indicate significant differences.

Next, among these lipids detected in different stages of pigs, the top 5 classes were represented as following: triacylglycerols (TGs), phosphatidlycholines (PCs), sphingomyelins (SMs), (DG), and (ChE) (Supplementary Figures S2A–C). PLS-DA analysis of differentially altered lipids (DALs) indicated that serum lipid profiles were dramatically shifted from Ad, through Ma to Oa pigs (Supplementary Figure S2D). To determine the temporal characteristics of the complete lipidomic dataset, Mfuzz was used for clustering analysis of 1,087 DALs across different stages of age, which divided all the DALs into 4 clusters (Figure 4A and Supplementary Table S8). As shown in Figure 4B, Cluster 1 and Cluster 4 were presented with an increasing trend in Ma and Oa groups when compared to the Ad group. We next found the DALs in Cluster 2 were exhibited a progressively declined in over the course of aging. However, it is also noteworthy that the levels of DALs in Cluster 3 were all dramatically decreased in Ma group. We then found that 35.98, 14.29, and 12.17% of DALs in Cluster 1 belongs to the lipid classes of triglyceride (TG), phosphatidylcholine (PC), and diradylglycerols (DG), respectively (Figure 4C). Within Cluster 2, 9.13% of DALs are TG, 19.8% are sphingomyelins (SM), 16.89% are ceramide (Cer), and 6.59% are PC (Figure 4D). These DALs were gradually decreased over age, suggesting that these DALs were specific diminished in response to the physiological alteration in relating to aging. The lipidomic analysis also showed that 20.82% of DALs in Cluster 3 were TG, and 17.14% were SM (Figure 4E). Additionally, when compared to pigs in Ad and Ma groups, swine in Oa was more profoundly in increasing the abundances of DALs in Cluster 4, in which 18.89% were TG, 27.42% were PC, and 13.13% were DG (Figure 4F). Furthermore, paired comparisons revealed 32 lipids exhibited significant differences between the Oa and Ma group, whereas 82 and 67 features showed obvious differences between the Ad versus the Ma and the Oa group, respectively (FDR <0.05, Supplementary Table S9). Of these significantly changed lipids, we screened out three lipids with significant differences in expression among the three groups (Figure 4G). The abundance of these three lipids were displayed in Figures 4H–J.

### 3.5. Summary of age-associated changes in microbial genes through KO genes and KEGG pathway modules

**Figure 5:**
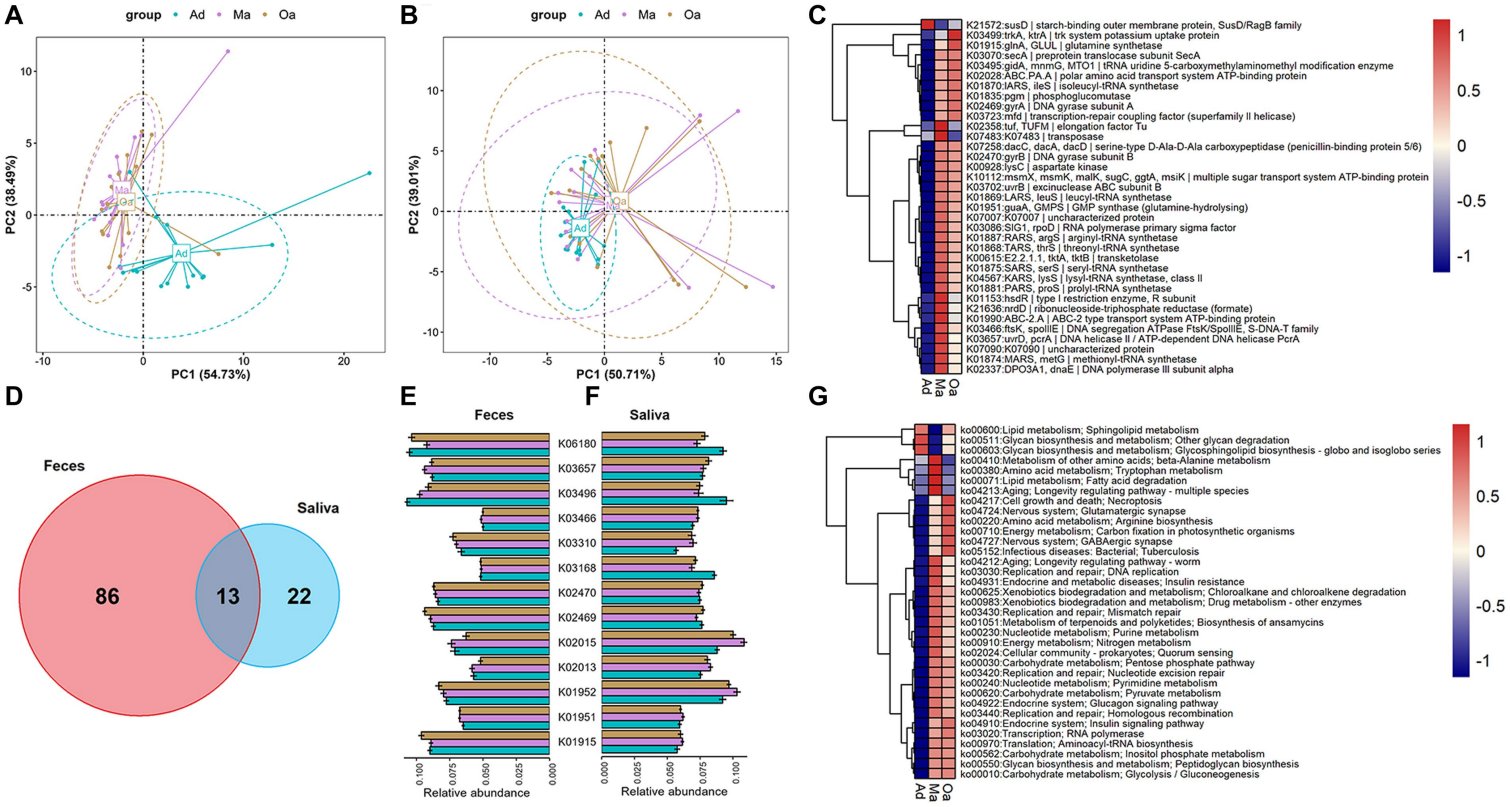
The alterations in the KO functional genes and pathways of the oral-gut microbiome that occur with aging. **(A,B)** Principal component analysis (PCA) based on KO genes and pathways of the microbiome in stool revealed a distinct separation trend between different groups. **(C)** The heatmap displays the abundance of differentially expressed KO genes in the microbiome of fecal samples across three groups. The abundance of each KO gene is represented by the mean value of the samples in each group. **(D)** A Venn diagram illustrates the intersection of differentially expressed KO genes in fecal samples across three groups and differentially expressed KO genes in saliva samples across three groups. **(E,F)** The bar chart (with error bars) displays the abundance of 13 differentially expressed KO genes that are present in both fecal **(E)** and saliva samples **(F)**. **(G)** The heatmap displays the abundance of differentially expressed KO pathways in the microbiome of fecal samples across three groups. The abundance of each KO pathways is represented by the mean value of the samples in each group.

Considering the multi-omics shift of the gut microbiome and metabolome with age, we hypothesized that metabolite and lipid differences might reflect differences in microbial enzyme gene expression. To further determine the microbial metabolic processes occurring in swine at different stages of age, we annotated metagenome-analysed microbial genes in the KEGG orthology (KO) database. Firstly, PCA analyses based on KO genes and functional ko pathways in faeces and saliva samples displayed a significantly different distribution of KO genes among three groups (Figures 5A,B; Supplementary Figures S3A,B). To examine the effect of age on influencing the prevalence of microbial enzymatic genes, we compared the relative abundance of KO genes among the three groups and between each of two groups. Notably, we observed a sum of 99 KO genes within the intestinal microbiota were showed significantly different in abundance in swine among three groups (Figure 5C and Supplementary Table S10). Among the differentially abundant genes, *gluA* and *pgm* gene abundance showed a tendency to rise gradually with increasing age. In the saliva samples of swine, 35 differentially expressed KO genes encoded by oral microbiota were detected (Supplementary Figure S3C and Supplementary Table S11), such as *glnA*, which dominant in Oa group, but had the lowest abundance in Ma group. Intriguingly, we found 13 discrepant KO genes that were intersected in the intestinal and salivary microbiota (Figure 5D). For instance, K03496 and K06180 were simultaneously enriched in the gut and salivary microbiome of Ad group. Overall, these intersecting differential genes are not similarly enriched across groups (Figures 5E,F). In the KO map analyses of gut microbial metabolism in the three stages of age, which revealed that the abundance of ko04217 (cell growth and death; necroptosis), ko05152 (infection disease; bacterial; tuberculosis) and ko04727 (nervous system; GABAergic synapse) were progressively increased from Ad group, through Ma group, to Oa group (Figure 5G and Supplementary Table S12). Among the differentially abundant pathways in salivary microbiota, we identified the dominant KEGG pathways that were enriched in the Oa group as the following, including ko01523 (drug resistance: antineoplastic; antifolate resistance), ko04211 and ko04213 (aging: longevity regulating pathway), and ko05134 (infectious diseases: bacterial; legionellosis), while amino acid metabolism (ko00200, ko00430, ko00471), cofactors and vitamins (ko00670), as well as Carbohydrate metabolism (ko00053, ko00520, ko00052) were mainly increased in Ma group (Supplementary Figure S3D and Supplementary Table S13). Furthermore, Venn diagram analysis revealed that 26 discriminatorily abundant ko maps that were present in both the oral and gut microbiota (Supplementary Figure S3E), of which the Pyruvate metabolism (ko00620) was increased progressively, and Peroxisome (ko04146) was declined gradually from Ad to Ma and then to Oa group (Supplementary Figures S3F,G).

### 3.6. The association between metabolites, lipids, and oral-gut microbiome in swine at different stages of age

**Figure 6:**
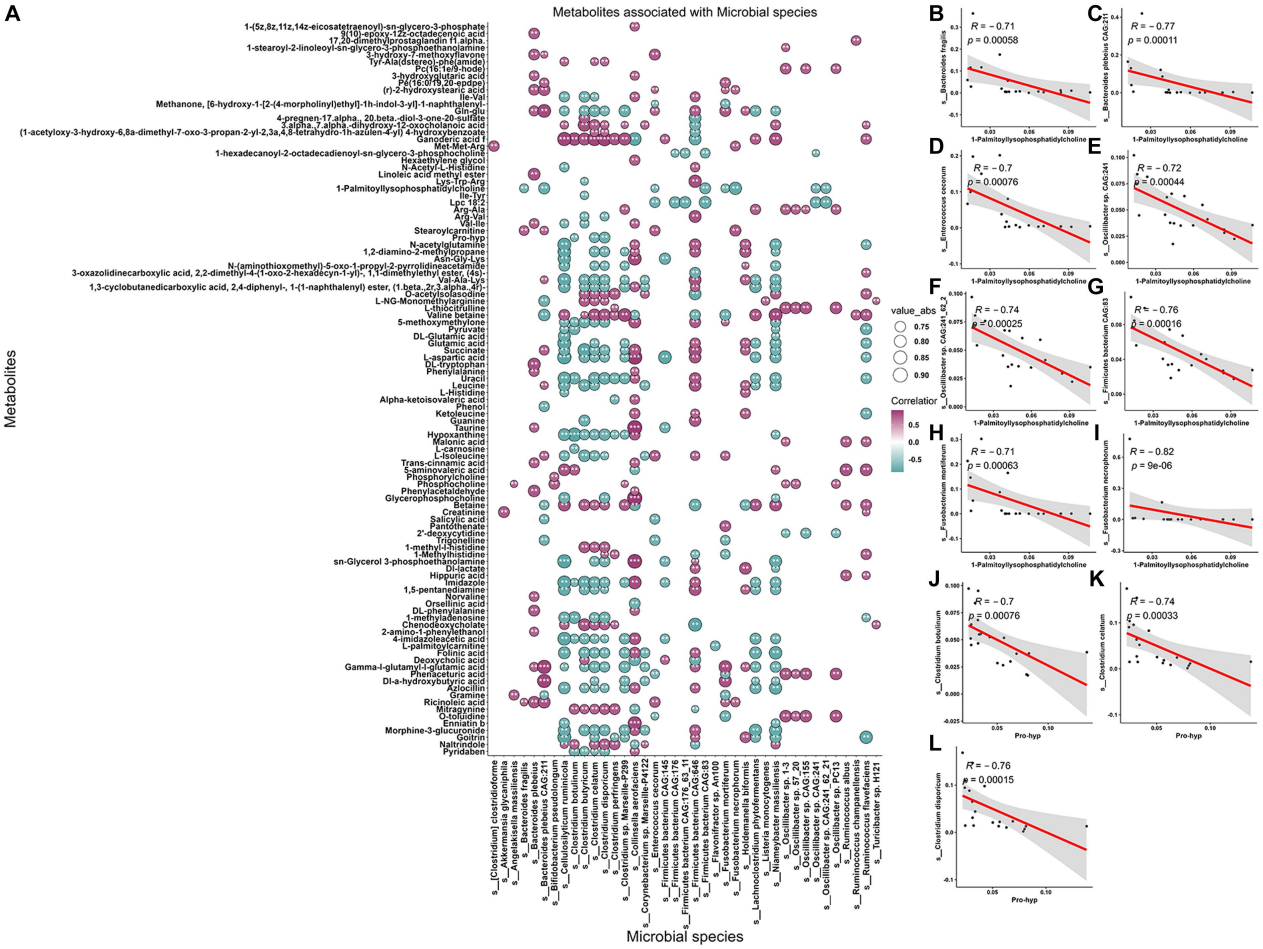
Association between metabolites, and gut microbiome of the swine in Ad and Ma groups. **(A)** The correlations between fecal microbes and serum metabolites were calculated. The absolute correlation coefficient (|*r*|) is represented by the size of the circle, and the adjusted *p*-value is indicated by an asterisk (“^*^”, *p* < 0.05, “^**^”, *p* < 0.01, “^***^”, *p* < 0.001). **(B–I)** Scatter plot representing the relationship between 1-palmitoyl-lysophosphatidylcholine and *Bacteroides fragilis*, *Bacteroides plebeius* CAG:211, *Enterococcus cecorum*, *Oscillibacter* sp. CAG:241, *Oscillibacter* sp. CAG:241_62_21, *Firmicutes bacterium* CAG:83, *Fusobacterium mortiferum*, and *Fusobacterium necrophorum*, respectively, by using Spearman rank sum test. **(J–L)** The relationship between pro-hyp and *Clostridium botulinum, Clostridium celatum, and Clostridium disporicum* was analyzed using a scatter plot and Spearman rank sum test.

The present investigation employed an intrinsic multi-omics analysis to elucidate the microbial features present in saliva, stool, serum metabolites, and lipids, demonstrating varied expression patterns among pigs at different age stages. Moreover, an association analysis was performed to comprehend the interplay between the altered oral-gut microbiota and the differentially abundant serum metabolites and lipids, utilizing Spearman rank correlation analysis. This analytical strategy facilitated the identification of co-varying features that relied on their mutual covariation with the ageing process. These findings provide valuable insights into the intricate interactions among diverse biological systems while highlighting potential avenues for further research in this domain. Notably, we observed significant disparities in the distribution of associations between paired microbial species and serum metabolites and lipids across various age stages. Specifically, when initially correlating these paired features to distinguish between pigs in the Ad group and Ma group, a multitude of positively and negatively associated features were detected (absolute coefficients >0.7 and FDR <0.05) (Figure 6A and Supplementary Table S14). Among the four metabolites that significantly discriminate between any two comparisons, it was observed that the abundance of several microbial species, such as *Bacteroides fragilis*, *Bacteroides plebeius* CAG:211, *Enterococcus cecorum*, *Oscillibacter* sp. CAG:241, *Oscillibacter* sp. CAG:241_62_21, *Firmicutes bacterium* CAG:83, *Fusobacterium mortiferum*, and *Fusobacterium necrophorum*, exhibited a negative correlation with 1-palmitoyl-lysophosphatidylcholine (Figures 6B–I). Similarly, species of *Clostridium botulinum*, *Clostridium celatum*, and *Clostridium disporicum* also exhibited an anticorrelation with the serum level of pro-hyp (Figures 6K,L). Additionally, a total of 161 associations between stool microbial species and serum lipids were identified (Supplementary Figure S4A and Supplementary Table S15). We further examined the association patterns between two lipids that significantly decreased from Ad, through Ma, to Oa, and stool microbes. It was observed that *Bacteroides fragilis*, *Bacteroides plebeius* CAG:211, and *Fusobacterium mortiferum* were positively associated with SM(d36:1) + HCOO (Supplementary Figures S4B,D). Interestingly, we also observed that the relative abundance of *Bacteroides fragilis*, *Bacteroides plebeius* CAG:211, and *Fusobacterium mortiferum* were positively correlated with SM(d38:4) + H (Supplementary Figures S4E–G). We also evaluated the associations between salivary microbes and serum metabolites and lipids, as described above. The number of correlations reached 301 and 476, respectively, by the time the association analysis was conducted between salivary microbes and serum metabolites and lipids (Supplementary Figures S5A, S6A and Supplementary Tables S16, S17). As shown in Supplementary Figures S5B–D, 1-palmitoyl-lysophosphatidylcholine was positively correlated with salivary *Kocuria* sp. ZOR0020, *Nigerium massiliense*, and *Staphylococcus sciuri*. Additionally, pro-hyp exhibited a positive association with *Flavobacterium columnare* and *Flavobacterium marinum* (Supplementary Figures S5E,F). Additionally, our investigation revealed that SM(d36:1) + HCOO declined with age and exhibited a negative correlation with eight salivary microbes. Notably, we observed a positive association between SM(d36:1) + HCOO and *Pelistega* sp. MC2 (Supplementary Figures S6B–J). Besides, we observed that SM(d38:4) + H exhibited an inverse correlation with four salivary microbes and a positive association with one salivary microbe (Supplementary Figures S6K–O). Furthermore, the results of the association analysis for paired microbiome, metabolites, and lipids in others any two groups are presented in Supplementary Tables S18–S25.

## 4. Discussion

Ageing is a significant risk factor for several age-related diseases, such as non-alcoholic fatty liver disease (NAFLD), type 2 diabetes mellitus, cardiovascular disease, neurodegenerative diseases, and cancer (Guo et al., 2022). To gain a better understanding of the ageing process, researchers have identified twelve hallmarks that contribute to this process. These hallmarks encompass genomic instability, telomere attrition, epigenetic alterations, loss of proteostasis, disabled macroautophagy, deregulated nutrient sensing, mitochondrial dysfunction, cellular senescence, stem cell exhaustion, altered intercellular communication, chronic inflammation, and dysbiosis. By comprehending these hallmarks and their underlying mechanisms, strategies can be developed to promote healthy ageing and, potentially, prevent or treat age-related diseases (Lopez-Otin et al., 2023). While the significant role of gut microbiota in influencing host health and disease has been well-established (Lynch and Pedersen, 2016), a comprehensive understanding of the intricate structure of the gut microbiome and the underlying mechanisms that interact with host aging in domestic pigs is still lacking. Therefore, the primary aim of this investigation is to discern the age-related alterations in both the oral and gut microbiome, as well as blood metabolites and lipids. By conducting this study under relatively uniform conditions, we seek to unravel the inter-relationships among these factors and shed light on the complex dynamics of host-microbiota interactions during the aging process.

In the current study, we investigated the effects of aging on oral-gut microbiome by utilizing multi-omics approaches, including shotgun metagenomics, LC-MS untargeted metabolomics and lipidomics analysis, and proteomics. In summary, our findings demonstrate that the elderly swine, particularly the Oa group, exhibited higher gut microbial diversity in both saliva and stool samples compared to the younger group living in the same farm, which in accordance with previous studies. Nevertheless, recent studies have revealed that centenarians are commonly associated with lower alpha diversity in their gut microbiome, as well as a decrease in butyrate-producing bacteria such as *Faecalibacterium*, *Roseburia*, and *Eubacterium*, instead with increased opportunistic pathogens (Biagi et al., 2017). Additionally, it is common for individuals to experience an upsurge in opportunistic pathogens within their gut microbiome, potentially due to a reduction in immune system function that results in a decrease in the population of beneficial gut bacteria. This decline in beneficial bacteria can lead to an amplification of opportunistic bacteria that may exert detrimental effects on health (Bosco and Noti, 2021). The observed increase in microbial diversity may signify a compensatory mechanism in response to the decline in beneficial gut bacteria, as the body endeavors to maintain a microbial equilibrium within the gut. Moreover, the greater alpha diversity observed in elderly individuals may be linked to longer colonic transit times and heightened exposure to environmental influences relative to younger individuals (Graff et al., 2001; Roager et al., 2016). Our research has revealed distinctive structural and functional traits of the gut microbiome in Oa pigs, highlighting an elevated abundance of specific beneficial bacterial species, including *Akkermansia*, in elderly swine. On the other hand, our study has demonstrated that aging is correlated with an increasing prevalence of pathogenic bacteria in the salivary microbiome, suggesting that alterations in the salivary microbiome may serve as a more precise indicator of the aging process within the body. Despite the clinical significance of gut microbiota composition, there is a paucity of research investigating the age-related dynamics of the microbiota, including changes in the F/B ratio. Only a limited number of researches have focused on examining the age-related changes in the F/B ratio in swine. This highlights the need for further research to better understand the dynamics of the gut microbiota throughout the aging process and its impact on host health and diseases. In this research, the F/B ratio was found to sharply increase from Ad to Ma, and then just as slightly decreased from Ma to Oa group, which exhibited controversy against previous reports (Makivuokko et al., 2010; Claesson et al., 2011; Kim et al., 2019). Research has shown that individuals with obesity and metabolic diseases typically have a higher F/B ratio, whereas those who are healthy have a lower ratio (Crovesy et al., 2020; Magne et al., 2020). Pigs belonging to the Oa group exhibited a higher F/B ratio compared to adult pigs, indicating dysbiosis in the gut microbiota of elderly swine, which may be linked to age-related diseases in pigs. Moreover, some studies have suggested that a higher F/B ratio could lead to increased energy harvest from the diet, subsequently resulting in weight gain, which may explain why pigs in the Ma group in the production phase require more energy to support their reproductive performance. During this period, sows generally gain weight and participate in reproductive and lactation activities, which necessitate greater nutrient intake, particularly energy. Therefore, further research is required to fully comprehend the intricate relationship between the F/B ratio and host aging.

Notably, it has been documented that aging is accompanied with chronic inflammation and inflamm-aging provided the possibility of studying aging-related diseases from a promising viewpoint (Kovtonyuk et al., 2016). 1-palmitoyl-lysophosphatidylcholine (1-PC) is reported as a bioactive lipid molecule that has been shown to play a role in inflammation (Hung et al., 2012; Liu et al., 2020). It is a type of lysophosphatidylcholine (LPC), which is a class of phospholipids that are involved in various physiological processes (Schmitz and Ruebsaamen, 2010; Law et al., 2019; Liu et al., 2020). Studies have found that 1-PC can activate immune cells such as macrophages and neutrophils, leading to the production of pro-inflammatory cytokines and chemokines (Liu et al., 2020). In addition, 1-PC has been shown to stimulate the production of reactive oxygen species (ROS), which can contribute to inflammation and tissue damage (Yoder et al., 2014). Furthermore, elevated levels of 1-PC have been observed in various inflammatory conditions such as atherosclerosis, and joint pain (Liu et al., 2020; Jacquot et al., 2022). In these conditions, 1-PC is thought to contribute to the pathogenesis of the disease by promoting inflammation and tissue damage. Overall, the available evidence suggests that 1-PC is involved in the regulation of inflammation and may contribute to the development and progression of various inflammatory conditions. The present study identified that the abundance of 1-PC was increased progressively with porcine age and negatively associated with gut *Bacteroides fragilis, Bacteroides plebeius CAG:211, Enterococcus cecorum*, but positively correlated with oral pathogenic *Staphylococcus sciuri*, and *Nigerium massiliense*. The observed increase in 1-PC levels in the bloodstream of elderly pigs may signify the manifestation of inflammaging, a chronic low-grade inflammatory state that is linked to aging. The upsurge in 1-PC may trigger the recruitment of lymphocytes and macrophages, resulting in the production of multiple inflammatory factors that can induce oxidative stress, amplify the level of inflammation in the body, and potentially fuel the onset of various age-related ailments. The correlation analyses revealed that there may be a complex interplay between these microbial species and this particular serum metabolite, which could potentially have implications for pig health and well-being. However, further research is needed to fully understand the mechanisms underlying these associations and their potential impact on pig health.

Pro-hyp is a dipeptide composed of proline and hydroxyproline that is found in collagen, a major component of connective tissues such as skin, tendons, and cartilage (Ohara et al., 2010; Ide et al., 2021). Collagen is produced by fibroblasts in various tissues throughout the body and is subsequently broken down into smaller peptides, predominantly pro-hyp, through various enzymatic processes (Ohara et al., 2007). Pro-hyp could be absorbed into circulation and transported to other tissues, where it exerts various beneficial effects, including anti-inflammatory, improve skin anti-decrepitude function and ameliorated joint condition (Zhang et al., 2010; Sontakke et al., 2016; Lee et al., 2019; Aguirre-Cruz et al., 2020). Osteoporosis is a disease characterized by low bone mass and structural deterioration of bone tissue, leading to increased bone fragility and a higher risk of fractures. Aging is a major risk factor for osteoporosis, as bone mass tends to decline with age. This is due to a combination of factors, including hormonal changes, decreased physical activity, and altered microbial composition. Studies have found that changes in the gut microbiota composition are associated with changes in bone density and structure. For example, some studies have found that the presence of certain bacterial species, such as *Lactobacillus*, is associated with increased bone density, while other species, such as *Prevotella*, are associated with decreased bone density. In our study, we discovered the declined level of pro-hyp in pigs progressively with age. In addition, we also found a significant negative correlation between decreased levels of pro-hyp and the abundance of gut *Clostridium botulinum*, *Clostridium disporicum* and *Clostridium celatum*, while showed a significant positive association with salivary *Flavobacterium columnare*, and *Flavobacterium marinum*. This could be due to the fact that gut bacteria, which are significantly negatively correlated with pro-hyp levels, increase in abundance with age, while oral microbiota, which are positively correlated with pro-hyp levels, decrease with age. In summary, the findings of this study suggest that supplementation with pro-hyp and probiotics that have the capacity to produce or process pro-hyp may represent a potential strategy for ameliorating age-related skeletal and skin aging. Notably, the interaction between pro-hyp and host microbiota shows promise as a novel avenue for the development of therapeutic interventions for aging-related disorders, including improved joint health and skin elasticity. These results have implications for the development of targeted therapies aimed at mitigating the negative effects of aging on the musculoskeletal and integumentary systems.

In summary, this investigation employed a unique approach by utilizing pigs residing in a controlled farm environment to mitigate confounding variables. The study revealed notable age-dependent modifications in both the structure and function of the oral and gut microbiota, as well as alterations in circulating levels of metabolites and lipids. Furthermore, we identified numerous interrelationships between perturbed microbial species and serum molecules, potentially influencing the maintenance of health and the development of diseases throughout different stages of host development and aging. However, the challenge of mitigating harmful inflamm-aging factors to promote healthy aging remains a significant obstacle. These findings hold significant implications for the advancement of therapeutic interventions and the progress of clinical applications targeting age-related diseases, utilizing pigs as a naturally aging animal model.

## Data availability statement

The original contributions presented in the study are publicly available. This data can be found here: NCBI SRA, PRJNA1015378.

## Ethics statement

The animal study was approved by All procedures were conducted according to the Regulations for the Administration of Affairs Concerning Experimental Animals (Ministry of Science and Technology, China, revised in March 2017), and approved by the Institutional Animal Care and Use Committee at the Experimental Animal Center of Hainan Academy of Agricultural Science (HNXMSY-20210503). The study was conducted in accordance with the local legislation and institutional requirements.

## Author contributions

GL and JZ conceived and designed the experiments and revised the manuscript. CQ, MH, and SW performed the experiments, analyzed the data, and wrote the manuscript. XJ, FW, XL, ST, ZC, WX, SG, JY, QL, XZ, and ZX collected the samples and performed the experiments. All authors contributed to the article and approved the submitted version.

## Funding

This work was supported by the grant from the Science and Technology Planning Project of Hainan Province (SQKY2022-0015), National Natural Science Foundation of China (Grant number 32260138), Project of Sanya Yazhou Bay Science and Technology City (Grant number SCKJ-JYRC-2022-97), Seed Laboratory of Yazhou Bay, Hainan Province (Grant number B22C11209), and UNDP-GEF Participatory *in*-*situ* conservation and sustainable use of agrobiodiversity in Hainan and Scientific Research of BSKY (XJ201922) from Anhui Medical University.

## Conflict of interest

The authors declare that the research was conducted in the absence of any commercial or financial relationships that could be construed as a potential conflict of interest.

## Publisher’s note

All claims expressed in this article are solely those of the authors and do not necessarily represent those of their affiliated organizations, or those of the publisher, the editors and the reviewers. Any product that may be evaluated in this article, or claim that may be made by its manufacturer, is not guaranteed or endorsed by the publisher.

## Footnotes

^1^https://www.mzcloud.org/

^2^http://www.metaboanalyst.ca
