# Supplementary material for: Multi-omics analysis reveals substantial linkages between the oral-gut microbiomes and inflamm-aging molecules in elderly pigs
Source: Front Microbiol. 2023 Sep 15;14:1250891. doi: 10.3389/fmicb.2023.1250891 (PMC10542583; doi:10.3389/fmicb.2023.1250891)
Supplement: Supplementary file 1 [file Data_Sheet_1.docx]

**Multi-omics analysis reveals substantial linkages between the oral-gut microbiomes and inflamm-aging molecules in elderly pigs**

Chuanmin Qiao^1, 2, 3#^, Maozhang He^4#*^, Shumei Wang^4#^, Xinjie Jiang^1^, Feng Wang^1^, Xin Jian Li^1^, Shuyi Tan^1^, Zhe Chao^1^, Wenshui Xin^1^, Shuai Gao^1^, Jingli Yuan^1^, Qiang Li^1^, Zichun Xu^1^, Xinli Zheng^1^, Jianguo Zhao^1*^, Guangliang Liu^1*^

**Supplementary figure legends**


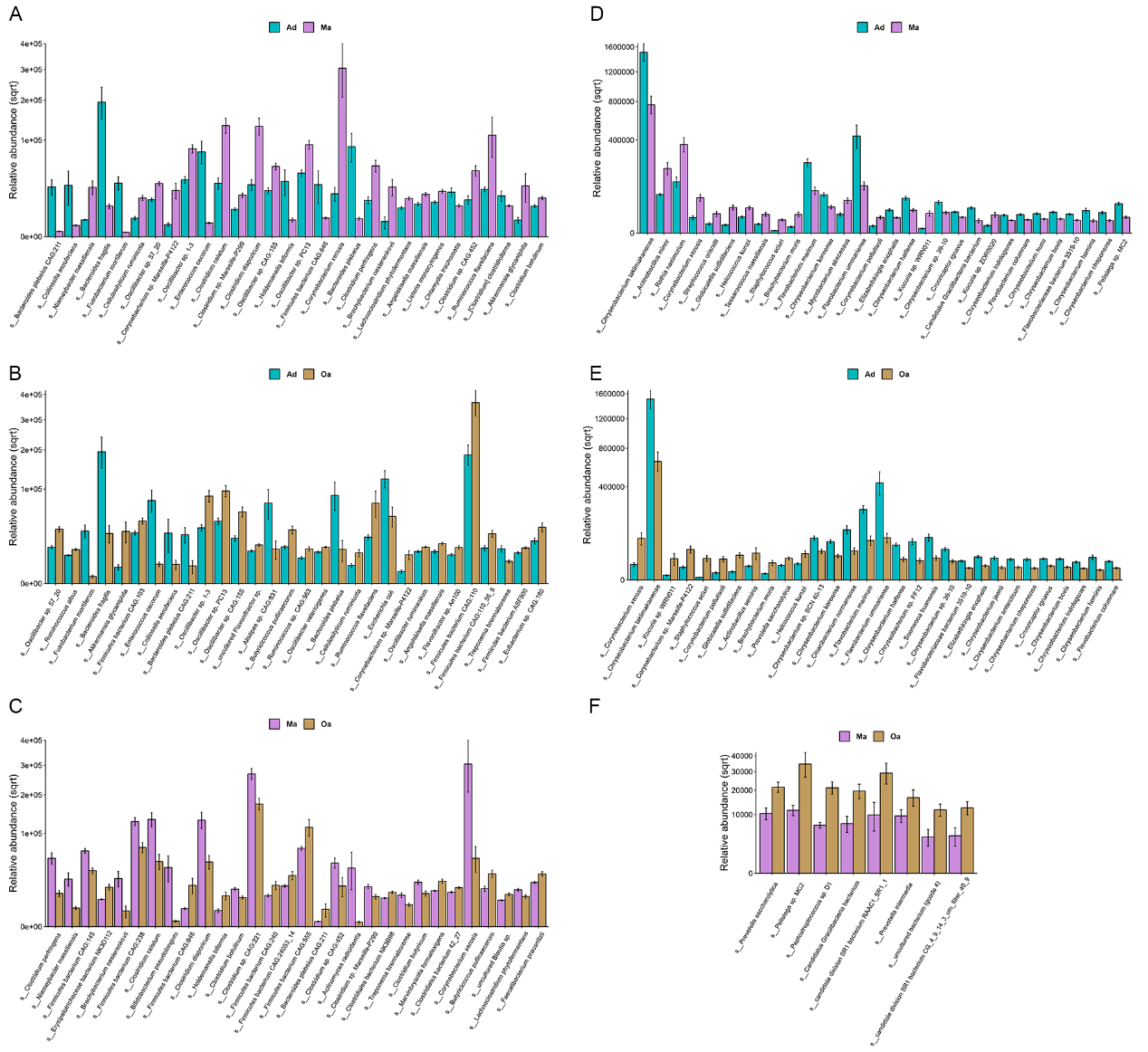


**Figure S1 Significant differences in fecal and salivary microbiota between any two age groups.** (A-C) The current research identified the top 30 microbial species that were significantly enriched or reduced in fecal specimens when comparing any two age groups (Ad, Ma, and Oa). (D-E) In comparison to Ad, our study has identified the top 30 microbial species that exhibit significant enrichment or reduction in both Ma and Oa groups, as evidenced by salivary samples. (F) Specifically, we have detected a total of eight salivary microbes at the species level that are significantly increased in the Oa group when compared to the Ma group.


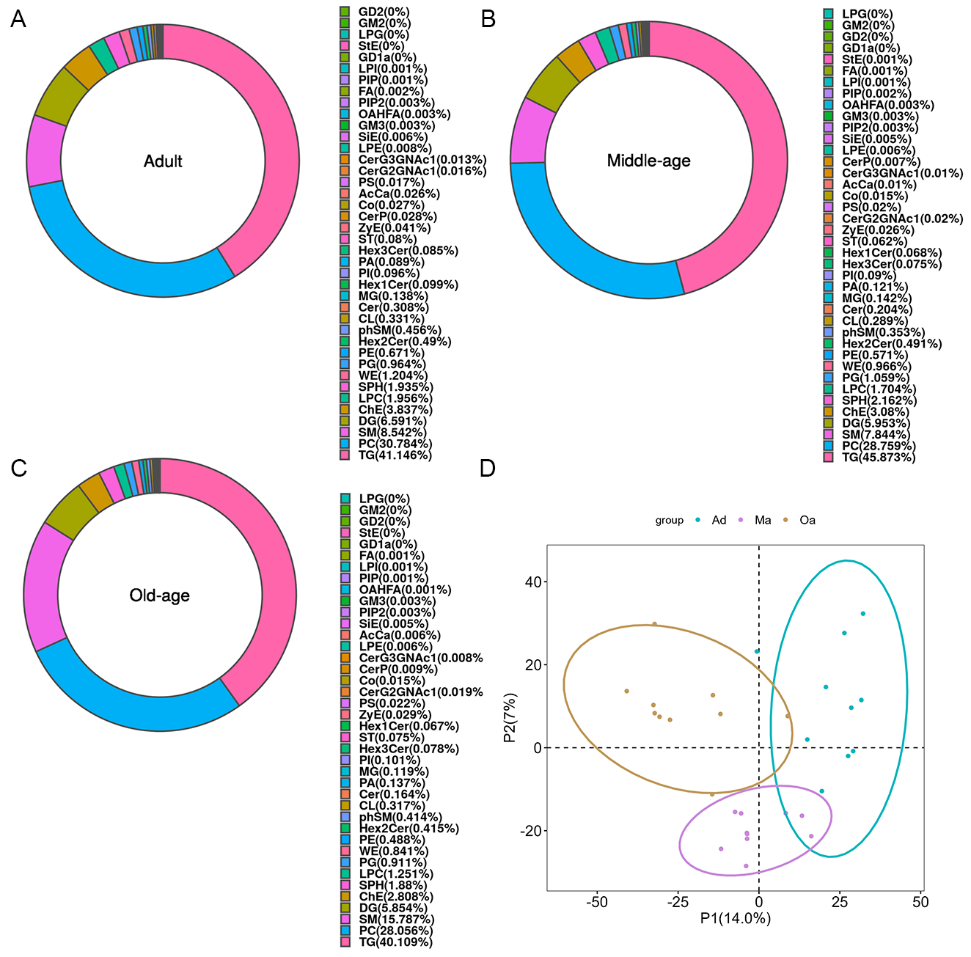
**Figure S2 Serum lipids identification and classification at class level.** (A) lipids of pigs in the stage of Adult. (B) lipids in the stage of Middle age. (C) lipids in the stage of Old age. (D) PLS-DA analysis based on differentially altered lipids (DALs) indicated that serum lipid profiles were dramatically shifted from Ad, through Ma to Oa pigs.


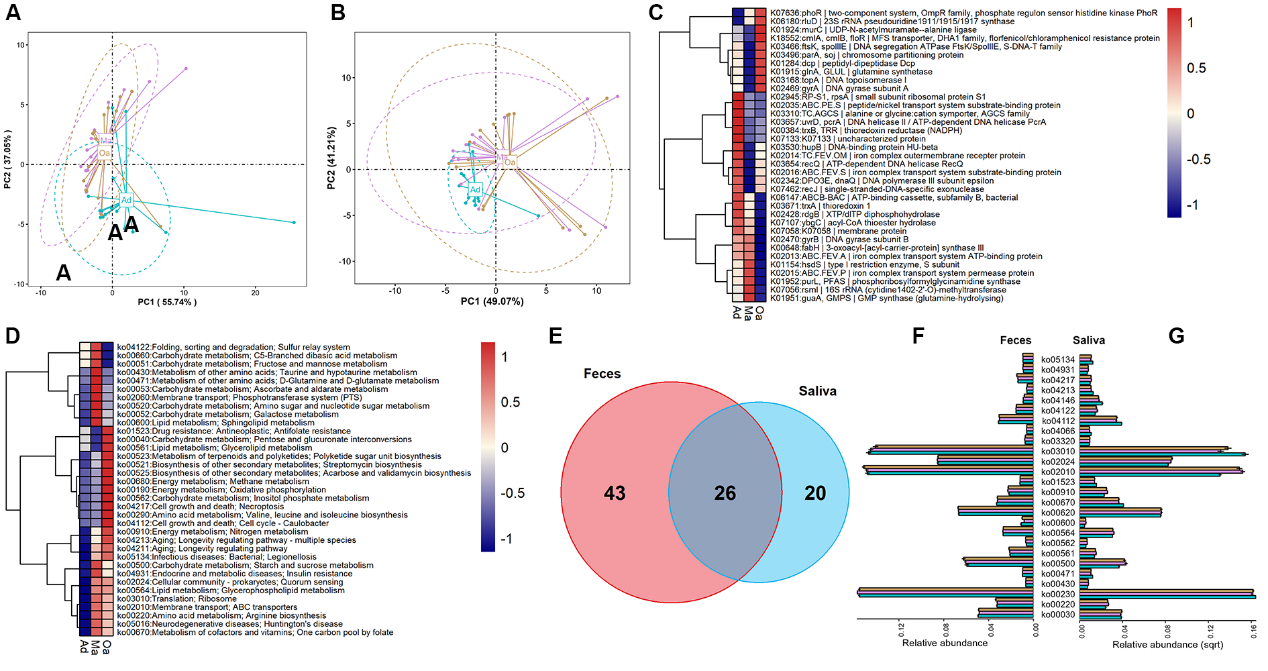


**Figure S3 Age-Associated Changes in Microbial Genes through KO Genes and KEGG Pathway Modules in salivary samples.** (A-B) Principal component analysis (PCA) based on KO genes and pathways of the microbiome in salivary samples revealed a distinct separation trend between different groups. (C) The heatmap displays the abundance of differentially expressed KO genes in the microbiome of sputum samples across three groups. The abundance of each KO gene is represented by the mean value of the samples in each group. (D) The heatmap displays the abundance of differentially expressed KO pathways in the microbiome of fecal samples across three groups. The abundance of each KO pathways is represented by the mean value of the samples in each group. (E) A Venn diagram illustrates the intersection of differentially expressed KEGG pathways in fecal samples across three groups and differentially expressed KO genes in saliva samples across three groups. (F-G) The bar chart (with error bars) displays the abundance of 26 differentially expressed KEGG pathways that are present in both fecal (F) and saliva samples (G).


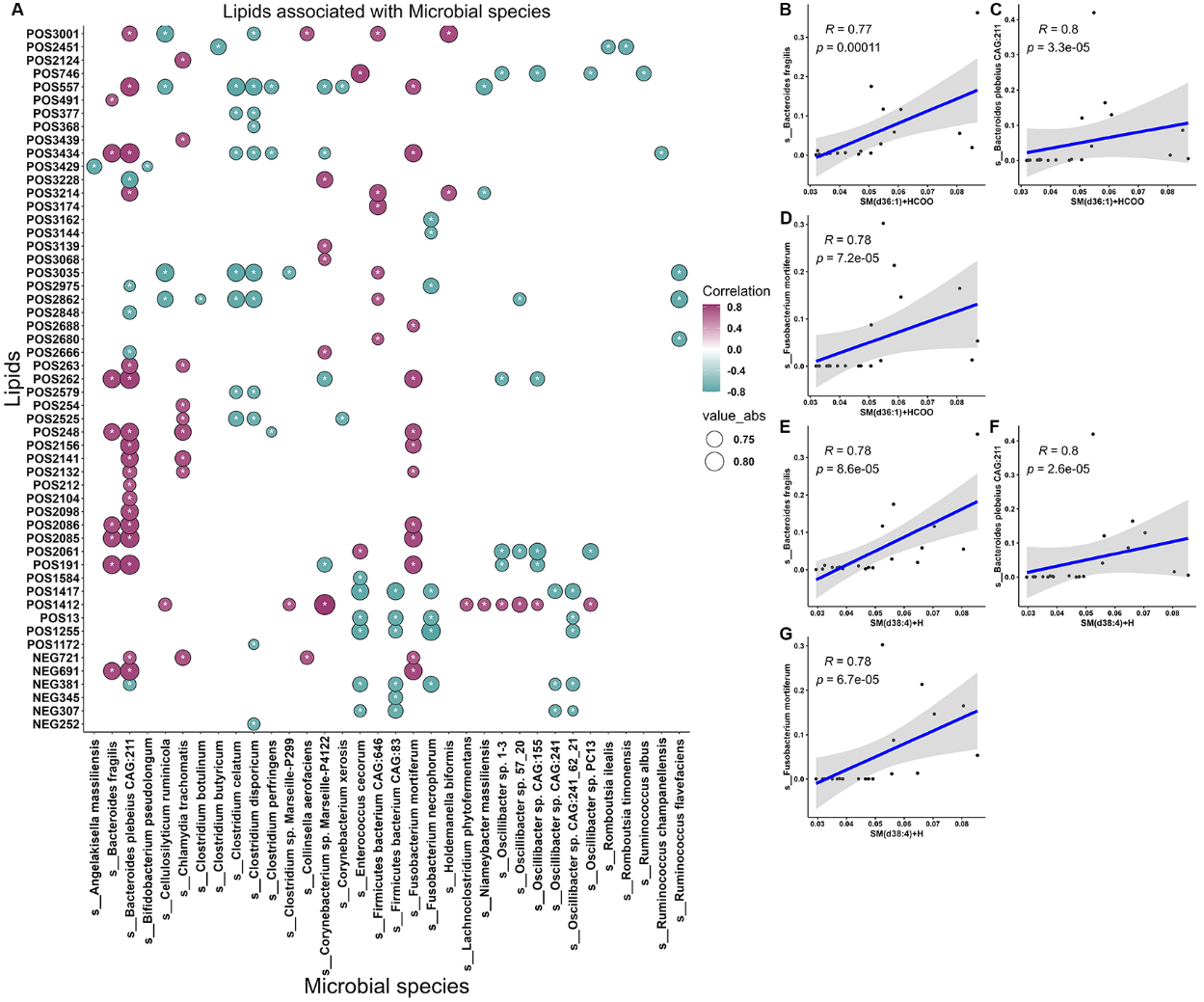


**Figure S4 Association between lipids, and gut microbiome** **of the swine in Ad and Ma groups.** (A) The correlations between fecal microbes and serum lipids were calculated. The absolute correlation coefficient (|r|) is represented by the size of the circle, and the adjusted P-value is indicated by an asterisk ("*", P < 0.05; "**", P < 0.01, "***", P<0.001). (B-D) Scatter plot representing the relationship between SM(d36:1)+HCOO and *Bacteroides fragilis*, *Bacteroides plebeius* CAG:211, and *Fusobacterium mortiferum*, respectively, by using Spearman rank sum test. (E-G) The relationship between SM(d38:4)+H and *Bacteroides fragilis*, *Bacteroides plebeius* CAG:211, and *Fusobacterium mortiferum* was analyzed using a scatter plot and Spearman rank sum test.


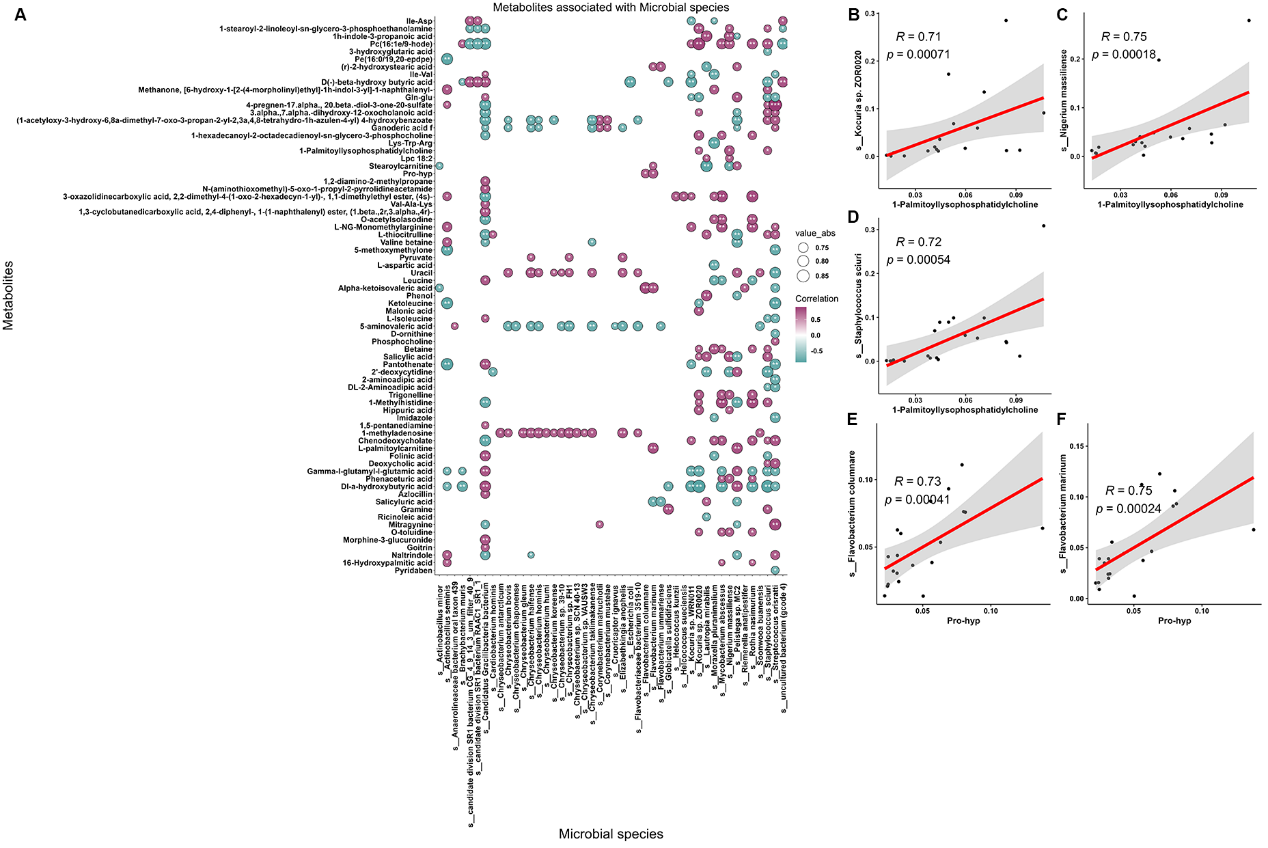
**Figure S5 Association between metabolites, and salivary microbiome of the swine in Ad and Ma groups.** (A) The correlations between salivary microbes and serum metabolites were calculated. The absolute correlation coefficient (|r|) is represented by the size of the circle, and the adjusted P-value is indicated by an asterisk ("*", P < 0.05; "**", P < 0.01, "***", P<0.001). (B-D) Scatter plot representing the relationship between 1-palmitoyl-lysophosphatidylcholine and *Kocuria sp. ZOR0020, Nigerium massiliense, and Staphylococcus sciuri*, respectively, by using Spearman rank sum test. (E-F) The positive relationships between Pro-hyp and *Flavobacterium columnare and Flavobacterium marinum* was identified using a scatter plot and Spearman rank sum test.


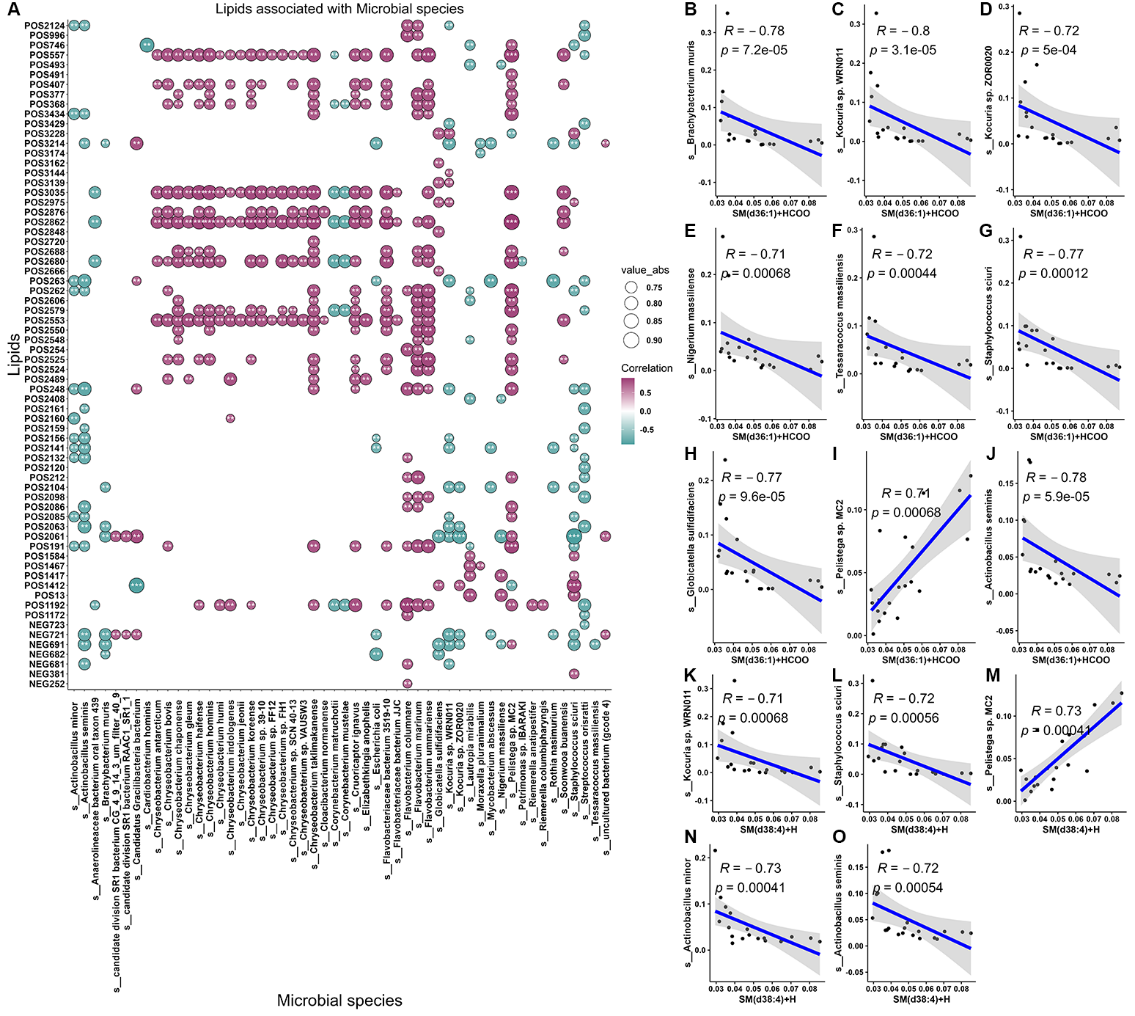


**Figure S6 Association between lipids, and salivary microbial species of the swine in Ad and Ma groups.** (A) The correlations between microbial species in the sputum samples and serum lipids were calculated. The absolute correlation coefficient (|r|) is represented by the size of the circle, and the adjusted P-value is indicated by an asterisk ("*", P < 0.05; "**", P < 0.01, "***", P<0.001). (B-J) Scatter plot representing the relationship between S SM(d36:1)+HCOO declined with age and exhibited a negative correlation with eight salivary microbes. Notably, we observed a positive association between SM(d36:1)+HCOO and *Pelistega sp.* MC2. (K-O) The relationship between SM(d38:4)+H exhibited an inverse correlation with four salivary microbes and a positive association with one salivary microbe.

**Supplementary Tables**

**Supplementary Table 1. Significantly different microbial species across three groups of pigs in faeces samples.**

**Supplementary Table 2. Microbial species in fecal samples that showed significant differences between any two groups of pigs.**

**Supplementary Table 3. Significantly different microbial species across three groups of pigs in salivary samples.**

**Supplementary Table 4. Microbial species in saliva samples that showed significant differences between any two groups of pigs.**

**Supplementary Table 5. Significantly differential metabolites between Ad and Ma groups.**

**Supplementary Table 6. Significantly altered serum metabolites between Ad and Oa groups.**

**Supplementary Table 7. Significantly altered serum metabolites between Ma and Oa groups.**

**Supplementary Table 8. Lipis classified into four clusters by Mfuzz package.**

**Supplementary Table 9. Significantly different lipids between different groups.**

**Supplementary Table 10. Significantly different KO genes in gut microbiome among three different age groups.**

**Supplementary Table 11. Significantly different KO genes in salivary microbiome among three different age groups.**

**Supplementary Table 12. Significantly different KEGG pathways in gut microbiome among three different age groups.**

**Supplementary Table 13. Significantly different KEGG pathways in salivary microbiome among three different age groups.**

**Supplementary Table 14. Relationships between significantly different gut microbes and metabolites in Ad and Ma groups.**

**Supplementary Table 15. Relationships between significantly different gut microbes and lipids in Ad and Ma groups.**

**Supplementary Table 16. Relationships between significantly different salivary microbes and metabolites in Ad and Ma groups.**

**Supplementary Table 17. Association analyses between significantly different salivary microbes and metabolites in Ad and Ma groups.**

**Supplementary Table 18-25. Correlation coefficients calculated between significantly altered oral-gut microbiome and serum metabolites and lipids when compared Oa with Ad groups, and Oa with Ma groups.**
